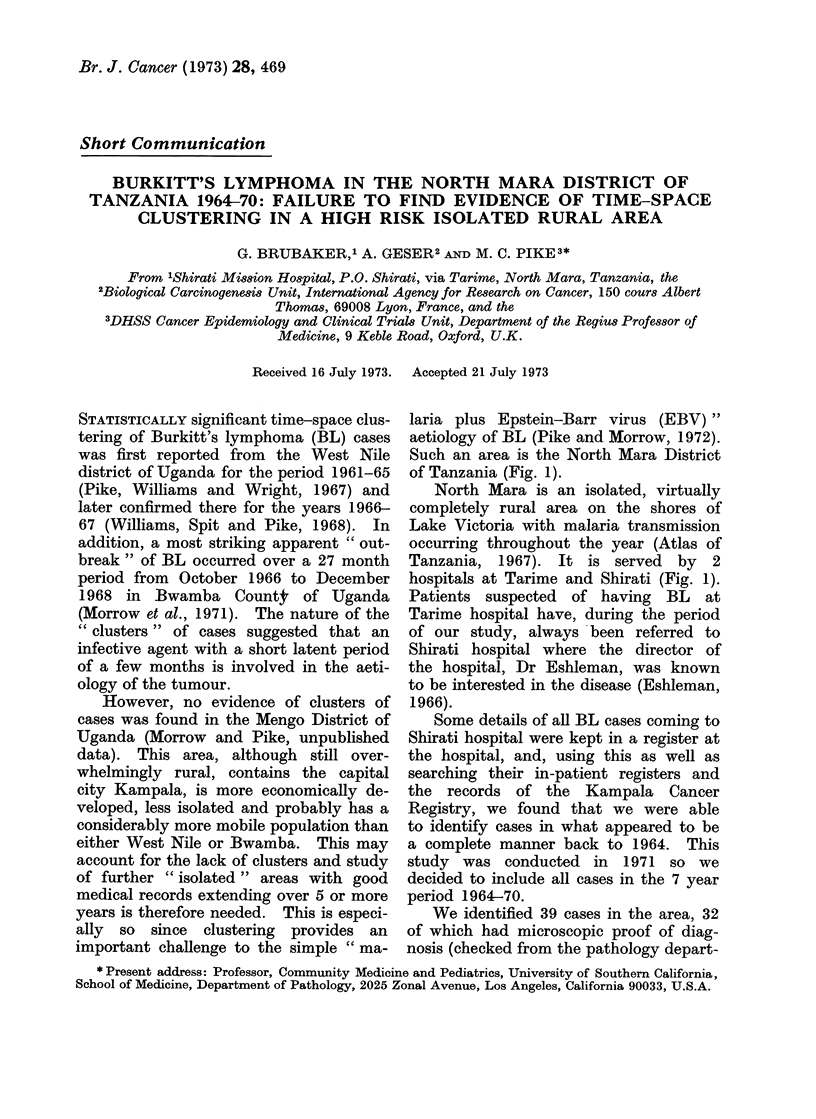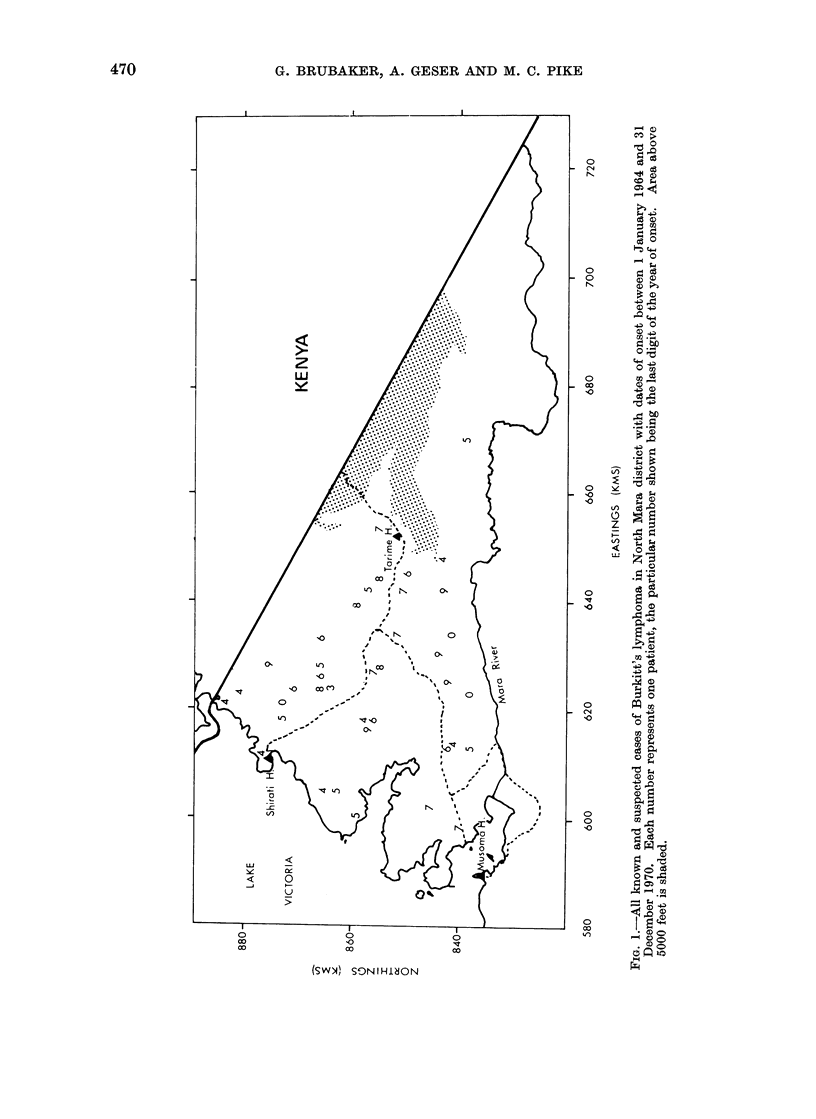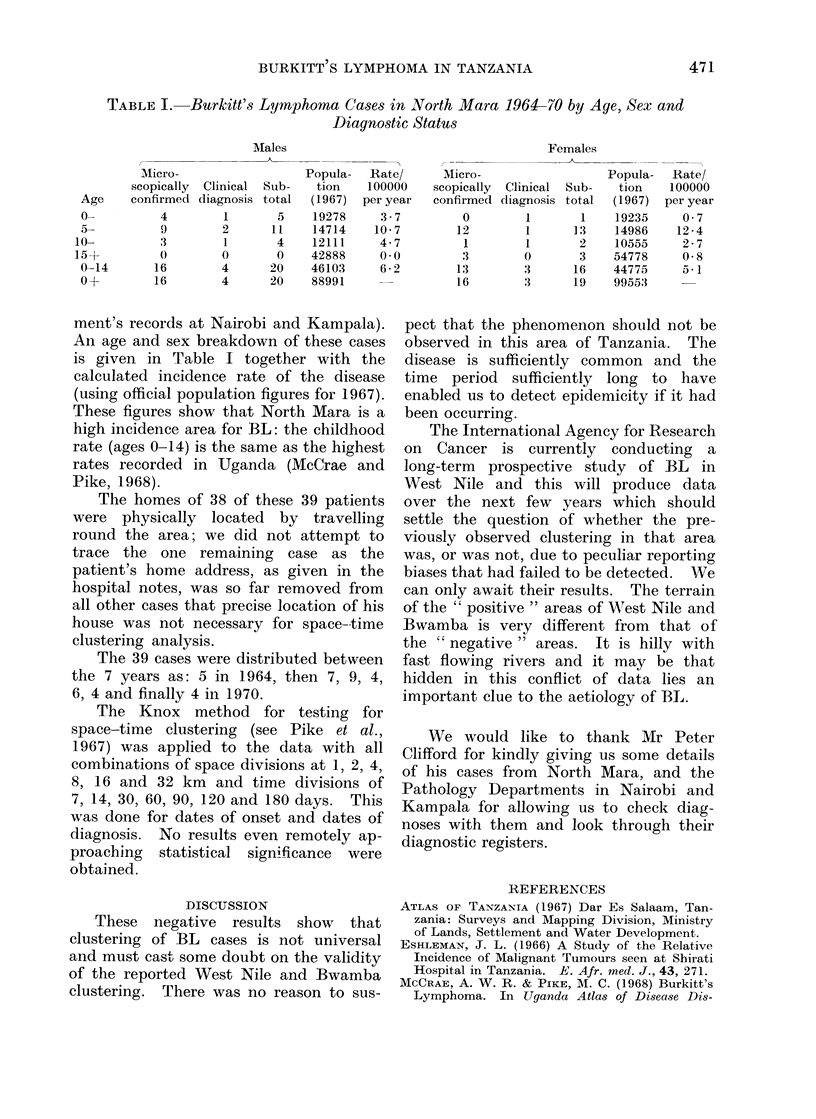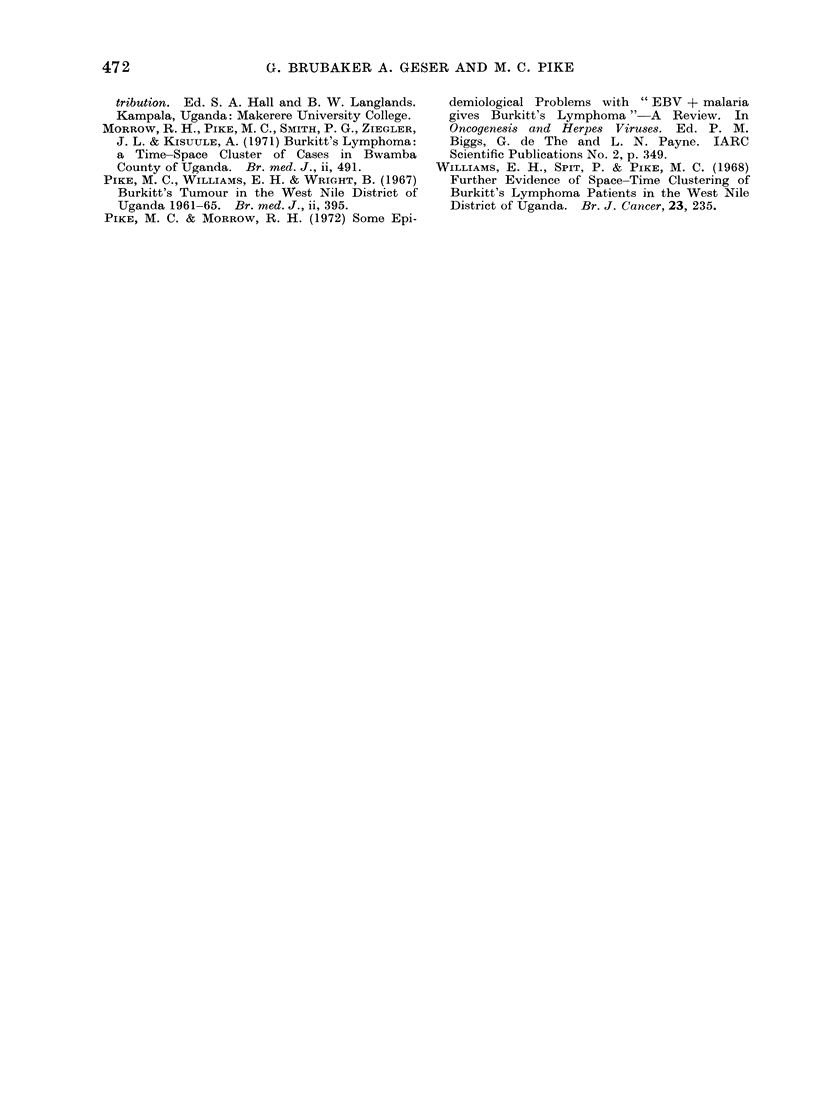# Burkitt's lymphoma in the North Mara district of Tanzania 1964-70: failure to find evidence of time-space clustering in a high risk isolated rural area.

**DOI:** 10.1038/bjc.1973.175

**Published:** 1973-11

**Authors:** G. Brubaker, A. Geser, M. C. Pike


*Br. J. Cancer* (1973) **28,** 469

*Short Communication*

### BURKITT'S LYMPHOMA IN THE NORTH MARA DISTRICT OF TANZANIA 1964–70: FAILURE TO FIND EVIDENCE OF TIME–SPACE CLUSTERING IN A HIGH RISK ISOLATED RURAL AREA

G. BRUBAKER,[1] A. GESER[2] AND M. C. PIKE[3]*

*From [1]Shirati Mission Hospital, P.O. Shirati, via Tarime, North Mara, Tanzania, the [2]Biological Carcinogenesis Unit, International Agency for Research on Cancer, 150 cours Albert Thomas, 69008 Lyon, France, and the [3]DHSS Cancer Epidemiology and Clinical Trials Unit, Department of the Regius Professor of Medicine, 9 Keble Road, Oxford, U.K.*

Received 16 July 1973. Accepted 21 July 1973

STATISTICALLY significant time–space clustering of Burkitt's lymphoma (BL) cases was first reported from the West Nile district of Uganda for the period 1961–65 (Pike, Williams and Wright, 1967) and later confirmed there for the years 1966–67 (Williams, Spit and Pike, 1968). In addition, a most striking apparent " outbreak " of BL occurred over a 27 month period from October 1966 to December 1968 in Bwamba County of Uganda (Morrow *et al.*, 1971). The nature of the " clusters " of cases suggested that an infective agent with a short latent period of a few months is involved in the aetiology of the tumour.

However, no evidence of clusters of cases was found in the Mengo District of Uganda (Morrow and Pike, unpublished data). This area, although still overwhelmingly rural, contains the capital city Kampala, is more economically developed, less isolated and probably has a considerably more mobile population than either West Nile or Bwamba. This may account for the lack of clusters and study of further " isolated " areas with good medical records extending over 5 or more years is therefore needed. This is especially so since clustering provides an important challenge to the simple " malaria plus Epstein–Barr virus (EBV) " aetiology of BL (Pike and Morrow, 1972). Such an area is the North Mara District of Tanzania (Fig. 1).

North Mara is an isolated, virtually completely rural area on the shores of Lake Victoria with malaria transmission occurring throughout the year (Atlas of Tanzania, 1967). It is served by 2 hospitals at Tarime and Shirati (Fig. 1). Patients suspected of having BL at Tarime hospital have, during the period of our study, always been referred to Shirati hospital where the director of the hospital, Dr Eshleman, was known to be interested in the disease (Eshleman, 1966).

Some details of all BL cases coming to Shirati hospital were kept in a register at the hospital, and, using this as well as searching their in-patient registers and the records of the Kampala Cancer Registry, we found that we were able to identify cases in what appeared to be a complete manner back to 1964. This study was conducted in 1971 so we decided to include all cases in the 7 year period 1964–70.

We identified 39 cases in the area, 32 of which had microscopic proof of diagnosis (checked from the pathology depart-

* Present address: Professor, Community Medicine and Pediatrics, University of Southern California, School of Medicine, Department of Pathology, 2025 Zonal Avenue, Los Angeles, California 90033, U.S.A.

FIG. 1.—All known and suspected cases of Burkitt's lymphoma in North Mara district with dates of onset between 1 January 1964 and 31 December 1970. Each number represents one patient, the particular number shown being the last digit of the year of onset. Area above 5000 feet is shaded.

TABLE I.—*Burkitt's Lymphoma Cases in North Mara 1964–70 by Age, Sex and Diagnostic Status*

| | Males | | | | | Females | | | | |
|---|---|---|---|---|---|---|---|---|---|---|
| Age | Micro-scopically confirmed | Clinical diagnosis | Sub-total | Popula-tion (1967) | Rate/ 100000 per year | Micro-scopically confirmed | Clinical diagnosis | Sub-total | Popula-tion (1967) | Rate/ 100000 per year |
| 0– | 4 | 1 | 5 | 19278 | 3·7 | 0 | 1 | 1 | 19235 | 0·7 |
| 5– | 9 | 2 | 11 | 14714 | 10·7 | 12 | 1 | 13 | 14986 | 12·4 |
| 10– | 3 | 1 | 4 | 12111 | 4·7 | 1 | 1 | 2 | 10555 | 2·7 |
| 15+ | 0 | 0 | 0 | 42888 | 0·0 | 3 | 0 | 3 | 54778 | 0·8 |
| 0–14 | 16 | 4 | 20 | 46103 | 6·2 | 13 | 3 | 16 | 44775 | 5·1 |
| 0+ | 16 | 4 | 20 | 88991 | — | 16 | 3 | 19 | 99553 | — |

ment's records at Nairobi and Kampala). An age and sex breakdown of these cases is given in Table I together with the calculated incidence rate of the disease (using official population figures for 1967). These figures show that North Mara is a high incidence area for BL: the childhood rate (ages 0–14) is the same as the highest rates recorded in Uganda (McCrae and Pike, 1968).

The homes of 38 of these 39 patients were physically located by travelling round the area; we did not attempt to trace the one remaining case as the patient's home address, as given in the hospital notes, was so far removed from all other cases that precise location of his house was not necessary for space–time clustering analysis.

The 39 cases were distributed between the 7 years as: 5 in 1964, then 7, 9, 4, 6, 4 and finally 4 in 1970.

The Knox method for testing for space–time clustering (see Pike *et al.*, 1967) was applied to the data with all combinations of space divisions at 1, 2, 4, 8, 16 and 32 km and time divisions of 7, 14, 30, 60, 90, 120 and 180 days. This was done for dates of onset and dates of diagnosis. No results even remotely approaching statistical significance were obtained.

### DISCUSSION

These negative results show that clustering of BL cases is not universal and must cast some doubt on the validity of the reported West Nile and Bwamba clustering. There was no reason to suspect that the phenomenon should not be observed in this area of Tanzania. The disease is sufficiently common and the time period sufficiently long to have enabled us to detect epidemicity if it had been occurring.

The International Agency for Research on Cancer is currently conducting a long-term prospective study of BL in West Nile and this will produce data over the next few years which should settle the question of whether the previously observed clustering in that area was, or was not, due to peculiar reporting biases that had failed to be detected. We can only await their results. The terrain of the "positive" areas of West Nile and Bwamba is very different from that of the "negative" areas. It is hilly with fast flowing rivers and it may be that hidden in this conflict of data lies an important clue to the aetiology of BL.

We would like to thank Mr Peter Clifford for kindly giving us some details of his cases from North Mara, and the Pathology Departments in Nairobi and Kampala for allowing us to check diagnoses with them and look through their diagnostic registers.

### REFERENCES

ATLAS OF TANZANIA (1967) Dar Es Salaam, Tanzania: Surveys and Mapping Division, Ministry of Lands, Settlement and Water Development.

ESHLEMAN, J. L. (1966) A Study of the Relative Incidence of Malignant Tumours seen at Shirati Hospital in Tanzania. *E. Afr. med. J.*, **43**, 271.

MCCRAE, A. W. R. & PIKE, M. C. (1968) Burkitt's Lymphoma. In *Uganda Atlas of Disease Dis-*

*tribution*. Ed. S. A. Hall and B. W. Langlands. Kampala, Uganda: Makerere University College.
MORROW, R. H., PIKE, M. C., SMITH, P. G., ZIEGLER, J. L. & KISUULE, A. (1971) Burkitt's Lymphoma: a Time–Space Cluster of Cases in Bwamba County of Uganda. *Br. med. J.*, ii, 491.
PIKE, M. C., WILLIAMS, E. H. & WRIGHT, B. (1967) Burkitt's Tumour in the West Nile District of Uganda 1961–65. *Br. med. J.*, ii, 395.
PIKE, M. C. & MORROW, R. H. (1972) Some Epidemiological Problems with "EBV + malaria gives Burkitt's Lymphoma"—A Review. In *Oncogenesis and Herpes Viruses*. Ed. P. M. Biggs, G. de The and L. N. Payne. IARC Scientific Publications No. 2, p. 349.
WILLIAMS, E. H., SPIT, P. & PIKE, M. C. (1968) Further Evidence of Space–Time Clustering of Burkitt's Lymphoma Patients in the West Nile District of Uganda. *Br. J. Cancer*, **23**, 235.